# Homophobia and heteronormativity as dimensions of stigma that influence sexual risk behaviors among men who have sex with men (MSM) and women (MSMW) in Lima, Peru: a mixed-methods analysis

**DOI:** 10.1186/s12889-019-6956-1

**Published:** 2019-05-21

**Authors:** Amaya G. Perez-Brumer, Ryan C. Passaro, Catherine E. Oldenburg, Jonathan Garcia, Jorge Sanchez, H. Javier Salvatierra, Javier R. Lama, Jesse L. Clark

**Affiliations:** 1 Columbia Mailman School of Public Health, Department of Sociomedical Sciences, 722 West 168th St., New York, NY 10032 USA; 2 College of Medicine, University of Tennessee Health Science Center, Memphis, TN USA; 3 South American Program in HIV Prevention Research, David Geffen School of Medicine, University of California Los Angeles, Los Angeles, CA USA; 4 Francis I Proctor Foundation, University of California, San Francisco, San Francisco, CA USA; 5 College of Public Health and Human Sciences, Oregon State University, Corvallis, Oregon USA; 6 Asociación Civil Impacta Salud y Educación, Lima, Peru; 7 Centro de Investigaciones Tecnologicas y Biomedicas Universidad Nacional de San Marcos, Lima, Peru

**Keywords:** Men who have sex with men and women (MSMW), Sexually transmitted infections (STIs), Human immunodeficiency virus (HIV), Partner notification, Social determinants of health

## Abstract

**Background:**

Stigma differentially influences HIV and STI care among MSM, especially regarding partner notification practices. Recognizing the heterogeneous behaviors/identities within the category “MSM,” we used mixed-methods to assess sexual risk behaviors among men who have sex with men only (MSMO) and behaviorally bisexual MSM (MSMW) with HIV and/or other STIs.

**Methods:**

MSMO/MSMW recently diagnosed (< 30 days) with HIV, syphilis, urethritis, or proctitis completed a cross-sectional survey assessing sexual risk behaviors, anticipated disclosure, and sexual partnership characteristics (*n* = 332). Multivariable generalized estimating equation models assessed characteristics associated with female compared to male partners in the last three partnerships. Follow-up qualitative interviews (*n* = 30) probed partner-specific experiences (e.g., acts and disclosure).

**Results:**

Among all participants, 13.9% (*n* = 46) described at least one of their last three sex partners as female (MSMW). MSMW (mean age of 31.8) reported a mean of 3.5 partners (SD = 4.5) in the past 3 months and MSMO (mean age 30.6) reported a mean of 4.6 partners (SD = 9.7) in the past 3 months. MSMW were more likely to report unprotected insertive anal sex (77.9%) than MSMO (43.1%; *p* < 0.01). Cisgender female partners were associated with condomless insertive sex in the last 3 months (aPR: 3.97, 95%CI: 1.98–8.00) and classification as a “primary” partnership (2.10, 1.34–3.31), and with lower prevalence of recent HIV diagnosis (0.26, 0.11–0.61). Planned notification of HIV/STI diagnoses was less common for female than for male partners (0.52, 0.31–0.85). Narratives illustrate internal (e.g., women as ‘true’ partners) and community-level processes (e.g., discrimination due to exposure of same-sex behavior) that position homosexual behavior and bisexual identity as divergent processes of deviance and generate vulnerability within sexual networks.

**Conclusions:**

MSMW recently diagnosed with HIV/STI in Peru report varying partnership characteristics, with different partner-specific risk contexts and prevention needs. Descriptions highlight how behaviorally bisexual partnerships cut across traditional risk group boundaries and suggest that HIV/STI prevention strategies must address diverse, partnership-specific risks.

## Background

Men who have sex with men (MSM) have 50 times the odds of HIV infection compared to the general population in Peru [1]. “MSM,” however, are a heterogeneous population and research in multiple international settings suggests that programs targeting gay-identified MSM may not be effective at reaching men who have sex with men and women (MSMW) [2–4]. While HIV prevalence among MSMW has been reported to be less than that among men who have sex with men only (MSMO), recent research shows that MSMW are less likely than their MSMO counterparts to access HIV prevention and testing services [2, 5]. They are also at higher risk for other sexually transmitted infections (STIs) than both MSMO and men who have sex with women only [4, 6–8].

Previous global studies have identified substantial heterogeneity within the category of “MSM,” documenting increased substance use, partner concurrency, and transactional sex in MSMW compared to MSMO [4–6, 9]. However, behavioral risk factors only tell a part of the story about how these men are exposed to STIs and HIV. Stigma has been described as a central mechanism increasing HIV vulnerability among gay, bisexual, and other men who have sex with men, all of whom violate societal expectations of gender roles and sexual behaviors, and often experience homoprejudice [10]. In heteronormative societies, where social, cultural, and institutional processes elevate heterosexuality as natural and normal and devalue other sexual orientations, non-gay identifying MSM may experience psychosocial stress over the prospect of being “outed” [11–13]. Similarly, as Peruvian sexual and gender norms are derived from social standards of *machismo*, heteronormativity may intersect with other forms of marginalization and stigma experienced by sexual/gender minorities to discourage disclosure of same-sex behavior by MSMW to their female partners [14, 15]. Accordingly, an in-depth understanding of sexual and gender politics, and how they inform partner expectations and disclosure practices, is critical to the development of successful interventions to promote sexual health among MSMW [15–18].

Stigma influences routine HIV care for all MSM, with MSMW less likely to access STI and HIV prevention programs targeted to MSM populations—usually due to fear of being identified as gay or homosexual [2, 18, 19]. Historically, stigmatizing language describing MSMW as a “bridge population” (i.e., responsible for transmitting STIs/HIV to their female partners) [16, 17] has had the unintended consequence of increasing psychosocial stress [6] and limiting the acceptability among MSMW of HIV prevention strategies targeted to gay men and transgender women (TW) [6]. However, little work has been done to address vulnerabilities particular to MSMW, which is crucial to increasing their access to prevention and treatment tools, and to reducing HIV infection within their sexual networks [20].

To address this gap and to provide an improved understanding of HIV/STI risk factors, we used mixed quantitative and qualitative methods to: 1) Describe similarities and differences in risk behavior and HIV prevalence between MSMO and MSMW, and 2) Assess and describe sex-specific partnership characteristics of sexual risk behavior among heterogeneous sexual identities and behaviors within the umbrella category of MSM recently diagnosed with HIV and/or an STI.

## Methods

### Study sample and procedures

All participants were recruited by staff at the *Asociación Civil Impacta Salud y Educación* [“Impact Health and Education” Civil Association] in Lima and the *Centro de Referencia de ITS* [STI Referral Center] “Alberto Barton” in Callao as part of a 2012 study of MSM and TW recently diagnosed with HIV and/or another STI. Participant recruitment, data collection methods, and data instruments have been previously described [21, 22]. Briefly, enrollment was limited to individuals who: 1) Were assigned male sex at birth, while some identified as cisgender men (gender identity congruent with natal sex) or as TW (assigned male sex at birth and reporting gender identity on a transfeminine spectrum), 2) Reported anal or oral intercourse with a male or TW partner during the previous year, and 3) Had been diagnosed within the previous 30 days with HIV, syphilis, genital herpes, urethritis or proctitis. TW were not included in the quantitative analytic sample for this manuscript due to the small number of those reporting female partners and partnerships.

Participants were invited to complete a survey about partner notification (PN) after completing post-test counseling, following Peruvian guidelines for standard PN recommendations. Participants were encouraged to complete the survey immediately after post-test counseling, but could return to complete the survey within a 30-day period, in order to accommodate the potential emotional distress surrounding an HIV/STI diagnosis. Despite having the option of returning at a later time, all subjects completed the survey at the time of diagnosis.

Interview participants were sampled by convenience, per participant and interviewer availability, and recruited from the larger quantitative study until reaching a point of qualitative data saturation. There were no statistically significant differences between the subset of participants interviewed and the larger study population. Interviews lasted 15–20 min and were audio-recorded, transcribed verbatim, and coded by two separate readers.

Written informed consent was obtained from all study participants prior to enrollment. The study protocol was reviewed and approved by the Ethics Review Board at the University of California (G10–03–036-01), Los Angeles (UCLA) and the Comite de Bioética [Bioethics Committee] at Asociación Civil Impacta Salud y Educación (0104–2010-CE). Participants were compensated 10 *Nuevos soles* ($4 USD) for their transportation costs.

### Measures

All participants completed an 82-item bio-behavioral, computer-assisted self-interview (CASI) survey addressing demographics, attitudes related to PN, and sexual practices with their three most recent sexual partners. Participants reported key characteristics of each partner, including relationship type and duration, their perception of the partner’s gender identity (male, female, transgender) and sexual orientation, and their perception of whether this partner was a likely source of their recently diagnosed STI. Partner-specific sexual acts were elicited, including type of intercourse (anal, vaginal, oral), sexual role during intercourse (insertive, receptive, both), and condom use during each reported act. Questions about likelihood of notifying their three most recent partners were measured via a 4-point Likert scale (Very Likely/Somewhat Likely/Somewhat Unlikely/Very Unlikely). Responses were re-categorized into binary (Likely/Unlikely) outcomes for analyses. Qualitative interviews followed a semi-structured script based on the main thematic groupings covered in the quantitative survey including, understandings of PN and how attitudes related to PN differed within the context of recent sexual practices and partnerships. Specifically, participants were asked to provide pseudonyms for their last three partners and to describe perceptions and rationale of anticipated PN. In-depth interviews were conducted with the purpose of exploring relationships within the thematic groupings in the cross-sectional survey in order to identify possible mechanisms to explore in future research.

### Statistical analysis

Bivariate comparisons using Fisher’s exact and Chi-Square tests for categorical variables (where appropriate) and t-tests for continuous variables compared characteristics between participants who described one or more of their last three partners as female (i.e., MSMW) and participants reporting only male partners (i.e., MSMO). All participants reporting either: 1) Only male partners; or 2) Both male and cisgender female partners were included in the multivariate analysis. Due to the small number of transgender women partners reported, TW partners were excluded from this analysis. The multivariable generalized estimating equation (GEE) model assessed participant- and partnership-level characteristics associated with female as compared to male partners in the three most recent partnerships. Variable selection for multivariable model used theoretical reasoning and substantive knowledge based on available literature [3–6, 14, 16, 21, 23]. Additionally, QIC subset analysis to assess best fit was conducted [24]. The GEE model was a binomial distribution and log link, with a sandwich estimator of the variance. All analyses were conducted in Stata 12.0 (StataCorp, College Station, TX).

### Qualitative analysis

Interviews (*N* = 30) were conducted after completing the quantitative survey in Spanish by a native Spanish -speaking interviewer, and audio recorded and transcribed verbatim. Guided by immersion crystallization, a qualitative approach that emphasizes both examining data and reflecting on the experience of being immersed in the data analysis, transcripts were analyzed based on an inductive and deductive approach to identify themes and relationships between themes. Initial sets of themes were independently assessed by two members of the research team who subsequently met to compare themes, discuss and reconcile any differences, and refine a set of codes and their definitions. A structured codebook was developed transforming the themes into codes. The coding scheme was applied to an initial transcript by two members of the team, and any discrepancies were discussed with a third member of the qualitative analysis group. All qualitative analysis was conducted using Dedoose Version 5.0.11 (Socio Cultural Research Consultants, LLC, https://www.dedoose.com. When qualitative data is presented in the results, participant ID (PTID), self-reported HIV status, and sexual orientation are reported.

## Results

Mixed-methods results are drawn from 332 participants who completed the bio-behavioral survey and the narratives of a subset of 30 participants. For quantitative data, 13.9% of 332 participants (*n* = 46) reported sex with at least one female partner and were classified as MSMW. MSMW often endorsed a bisexual (45.4%) or heterosexual (43.2%) identity, and reported a mean of 3.5 partners (SD = 4.5) in the past 3 months. Most MSMO identified as gay or homosexual (84.3%) and reported a mean of 4.6 partners (SD = 9.7) in the past 3 months. More than half of MSMO in our sample were recently diagnosed with HIV (51.4%), compared to 23.9% of MSMW (See Table 1).

**Table 1:** Individual and partnership-level characteristics of MSMO and MSMW in Lima (*N* = 332; 1073 partnerships)

	Any female partner in last 3 partnerships (*N* = 46)	No female partners in last 3 partnerships (*N* = 286)	Overall (*N* = 332)	*p*-value
Age (mean, SD)	31.8 (10.9)	30.6 (8.8)	30.7 (9.1)	0.793
Education (University or above vs. secondary or less)^a^	14 (30.4)	142 (49.8)	156 (47.1)	0.029
Sexual identity				
Heterosexual	19 (43.2)	4 (1.4)	23 (7.1)	< 0.001
Bisexual	20 (45.4)	40 (14.3)	60 (18.5)	0.001
Homosexual	5 (11.4)	236 (84.3)	241 (74.4)	
HIV/STI status				
STI only	35 (76.1)	139 (48.6)	174 (52.4)	
HIV	11 (23.9)	147 (51.4)	158 (47.6)	
Number of partners in last 3 months (mean, SD)	3.5 (4.5)	4.6 (9.7)	4.4 (8.9)	0.474
Partnership-Specific Characteristics				
	Female Partner (*N* = 86)	Male Partner (*N* = 987)	Overall (*N* = 1073)	*p*-value
Likely to disclose to partner				
No	38 (45.2)	331 (41.7)	369 (42.0)	0.62
Yes	41 (48.8)	390 (49.1)	431 (49.1)	
I don’t know	5 (5.6)	73 (9.2)	78 (8.9)	
Any unprotected insertive sex with partner in the last 3 months	67 (77.9)	348 (43.1)	415 (46.5)	< 0.001
Any oral sex with partner in the last 3 months	72 (83.7)	681 (84.4)	753 (84.3)	0.88
Partner is the primary partner	33 (38.4)	248 (30.7)	281 (31.5)	0.18

Fisher’s exact test was used to calculate *p*-values for categorical variables and t-tests for continuous variables

^a^n (%), unless otherwise noted

The quantitative survey also assessed partnership -specific characteristics of the last three sexual partners of each participant. Of 1073 sexual partnerships reported, 8.0% (*n* = 86) were with female partners. Across participants, men reporting female partners were more likely to report any unprotected insertive sex as compared to those reporting only male partners in the last 3 months: 77.9% versus 43.1% (*p* < 0.01).

In the qualitative data, 43.3% of participants were living with HIV (i.e., HIV-infected). Results below present quantitative relationships and qualitative findings to explore possible explanations for these associations. Notably, participant narratives highlight intersecting themes related to the pervasive role of stigma across MSMO and MSMW, and the heteronormativity embedded within MSMW partnerships.

Table 2 summarizes results from the multivariable model assessing factors associated with female partnerships. Participants with a female partner were less likely to have a college education (aPR, 95% CI: 0.46, 0.22–0.99) and less likely to test positive for HIV (0.26, 0.11–0.61). Participants had decreased likelihood of reporting they would disclose their HIV or STI diagnosis to their partner in female partnerships compared to male partnerships (0.52, 0.31–0.85). Multiple forms of stigma were discussed across the qualitative interviews as a reason for decreased likelihood to disclose a recent HIV and/or STI diagnosis. Participants noted that they would not disclose due to “fear of rejection” (PTID#59, HIV-positive, heterosexual) and “fear of being discriminated against” (PTID #56, HIV-negative, gay). Furthermore, “one always thinks that [if they disclose] they will be rejected. This is due to one’s life experiences, which lead one to fear and to be scared to say something” (PTID #18, HIV-positive, gay). A few participants voiced anxiety about an unknown and potentially violent reaction, as illustrated by one HIV-uninfected bisexual participant, “it’s a fear, a fear that we will lose contact, their friendship, or whatever else, it is a fear, a fear in their primordial response” (PTID #05, HIV-negative, bisexual).

**Table 2:** Behavioral factors associated with female partnerships among MSMO and MSMW in Lima (*N* = 227; 601 partnerships)

	aPR (95% CI)	*p*-value
	Participant Characteristics	
Age (years)	1.03 (0.99 to 1.06)	0.101
Education (University or above vs. secondary or less)	**0.46 (0.22 to 0.99)**	**0.049**
HIV/STI Status		
STI only	Ref	Ref
HIV	**0.26 (0.11 to 0.61)**	**0.002**
Number of unique male/ female partners in last month	0.93 (0.84 to 1.04)	0.205
	Partnership Characteristics	
Likely to disclose HIV/STI infection to partner		
No	Ref	Ref
Yes	**0.52 (0.31 to 0.85)**	**0.009**
I don’t know	1.84 (0.61 to 5.50)	0.278
Any unprotected penetrative sex with partner in the past 3 months	**3.97 (1.98 to 8.00)**	**< 0.001**
Any oral sex with partner in the past 3 months	0.52 (0.20 to 1.45)	0.212
Partner is primary sexual partner	**2.10 (1.34 to 3.31)**	**0.001**

*Abbreviations*: *aPR* adjusted prevalence ratio, *CI* confidence interval

*p* value ≤0.05 and bold text represents statistical significance

Specific to MSMW, fear of disclosure encompassed an additional dimension of homophobia. For example, when discussing why he would not disclose to his female partner, one participant stated, “for the fear that she will say, ‘What are you doing with men?” (PTID #71, HIV-positive, heterosexual). Another stated, “because she would ask me who I’d been with and I couldn’t tell her” (PTID #81, HIV-uninfected, bisexual). Notably, these sentiments were echoed by MSMO as well, who noted pervasive stigma rooted in homophobia in the perception of gay men as high-risk for HIV and other STIs, as stated: “Most heterosexuals think that only us gays have infections and that is a lie. They, those who are bisexual, have sex with both men and women. That is the reason women are also infected” (PTID#17, HIV-negative, gay).

Female partnerships were associated with an increased prevalence of unprotected insertive sex in the last 3 months (aPR = 3.97, 95% CI 1.98 to 8.00). Partnerships with women were also more likely to be classified as “primary” (2.10, 1.34 to 3.31). These findings were corroborated across the interviews. Men who reported having sexual relations with both men and women frequently described their female partners as “my actual partner, my true partner” (PTID #59, HIV-positive, heterosexual) and “she is my only partner, the others are friends” (PTID #64, HIV-negative, heterosexual). Participant narratives frequently named a female partner as the primary partnership. For example, one self-identified gay participant who reported only having sex with other men (MSMO) described his experience with a behaviorally bisexual partner:

“I think he suffered because he enjoyed what we did but I think he got tired of it and now only likes women…Within his community they found out about us and they punished him, and now he doesn’t spend time with homosexual people. You understand?” (PTID #73, HIV negative, gay).

## Discussion

Our findings demonstrate that about 14% of the sample reported both male and female sexual partners, with specific partner-level factors (e.g., decreased disclosure of HIV/STI diagnosis and lower likelihood of condom use for insertive sex) associated with female sexual partnerships. By accounting for two levels of quantitative analyses (individual and partnership) and incorporating mixed-methods, these results highlight the unique risks for behaviorally bisexual MSM and their varying partnership characteristics. Perspectives of MSMO and MSMW highlight the pervasive influence of heteronormativity and homophobia in their sexual risk-taking and partner notification. Jointly, these results underscore the need to recognize sexual diversity within the category of “MSM” and to incorporate strategies into STI and HIV prevention that do not unintentionally exclude stigmatized and/or hidden subgroups, like those of non-gay identifying behaviorally bisexual men.

At the individual level, our sample exhibited a high heterogeneity of sexual identity, with almost 50% of MSMW identifying as heterosexual. Qualitative results emphasize the centrality of heterosexual identity for many MSMW, and suggest a community-wide perception that their identity may put them at lower risk for HIV infection, regardless of their same-sex sexual risk behavior. This finding parallels existing literature [18], and underscores the difficulty of successfully directing STI and HIV prevention resources to this high-risk population frequently described as “hidden” in the interstices between traditional heterosexual and homosexual networks. Furthermore, mixed-methods results evidence the internal and community-level processes that position homosexual practices as aberrant and bisexual identity as deviant, though differentiating the two as separate categories of socially unacceptable behavior. It is against this broader backdrop that barriers to disclosure of recent HIV and STI diagnosis and differential partnership status between female and male partners must be understood.

Previous studies have detailed behavioral vulnerabilities of MSMW [23, 25]. We extend this literature by exploring partner-level factors and individual experiences to assess how MSM maintain different sexual risk behaviors with female and male partners. The men in our sample were more likely to report condomless insertive sex with female compared to male partners, consistent with the literature [2]. Peruvian MSM are more likely to engage in condomless anal intercourse with stable partners, at least partially as a result of the association of condomless sex with fidelity and trust [21]. In this case, females were more likely to be considered primary partners and primary partnerships with females were associated with a higher frequency of condomless intercourse. Qualitative results supported this finding as MSMW reported hiding their same-sex sexual behavior, and as a result concealing their HIV/STI diagnoses, from their “true,” or female, partners. While men with female partners had a lower prevalence of HIV than those with only male partners, the overall prevalence of HIV in our sample of MSMW (23.9%) was much higher than the 0.3–0.5% prevalence observed in the general adult male Peruvian population [1]. Previous analyses of this same population also found a lower likelihood of notification among men diagnosed with HIV compared to those with other STIs, suggesting that HIV-specific notification would be even lower in this population with already low frequencies of informing female partners of their potential STI exposure [26]. Given the key role of partner notification in network-based approaches to HIV and STI prevention, efforts to address the behavioral, cultural, and structural barriers to notification of both male and female partners of MSMW are a critical area for future research on population-level approaches to combined HIV-STI prevention.

Our findings should be considered in the context of several limitations. First, results may not be generalizable to all MSM in Lima due to the convenience sampling methods employed. As a recent STI or HIV diagnosis was a criterion for enrollment, our sample is likely to be higher risk, and to have a higher prevalence of HIV and STIs, compared to the general MSM population. Second, the risk profile of MSMW presenting for testing and treatment services at a center for STI/HIV research associated with the MSM community and a municipal STI clinic may be different than that of other MSMW. Finally, our study was cross-sectional and causation cannot be inferred from the observed associations.

## Conclusion

Our study advances knowledge on the heterogeneity of sexual risk behaviors and identities among MSM by providing individual and partner-level factors associated with bisexual behavior among individuals recently diagnosed with HIV and other STIs. Our results account for the unique perspectives of the community regarding their sexual relationships with male and female partners, highlighting the roles of homophobia and heteronormativity in sexual risk practices and partner notification behavior. MSMW recently diagnosed with HIV/STI report varying partnership characteristics with different associated risk contexts and prevention needs. Given the extent to which their partnerships cut across traditional risk group boundaries, developing prevention strategies that successfully understand and address the diversity of their partnership-specific risk settings may significantly impact the spread of HIV/STIs in Lima, Peru.

## Abbreviations

AIDS: Acquired immunodeficiency syndrome
aPR: Adjusted prevalence ratio
CI: Confidence interval
GEE: General estimating eq.
HIV: Human immunodeficiency virus
MSM: Self-reported men who have sex with men
MSMO: Self-reported men who have sex with men only
MSMW: Self-reported men who have sex with men and women
PTID: Participant identification code
SD: Standard deviation
STI: Sexually transmitted infections
TW: Self-reported transgender women
